# Proteasome Activity Is Affected by Fluctuations in Insulin-Degrading Enzyme Distribution

**DOI:** 10.1371/journal.pone.0132455

**Published:** 2015-07-17

**Authors:** Diego Sbardella, Grazia Raffaella Tundo, Francesca Sciandra, Manuela Bozzi, Magda Gioia, Chiara Ciaccio, Umberto Tarantino, Andrea Brancaccio, Massimo Coletta, Stefano Marini

**Affiliations:** 1 Department of Clinical Sciences and Translational Medicine, University of Rome Tor Vergata, Via Montpellier 1, I-00133, Rome, Italy; 2 Center for Space Biomedicine, University of Roma Tor Vergata, Via Montpellier 1, I-00133 Roma, Italy; 3 Istituto di Chimica del Riconoscimento Molecolare (CNR), Università Cattolica del Sacro Cuore, Largo F. Vito 1, I-00168, Rome, Italy; 4 Istituto di Biochimica e Biochimica Clinica, Università Cattolica del Sacro Cuore, Largo F. Vito 1, I-00168, Rome, Italy; 5 School of Biochemistry, Medical Sciences Building, University Walk, Bristol, B581TD, United Kingdom; University of Geneva, SWITZERLAND

## Abstract

Insulin-Degrading-Enzyme (IDE) is a Zn^2+^-dependent peptidase highly conserved throughout evolution and ubiquitously distributed in mammalian tissues wherein it displays a prevalent cytosolic localization. We have recently demonstrated a novel Heat Shock Protein-like behaviour of IDE and its association with the 26S proteasome. In the present study, we examine the mechanistic and molecular features of IDE-26S proteasome interaction in a cell experimental model, extending the investigation also to the effect of IDE on the enzymatic activities of the 26S proteasome. Further, kinetic investigations indicate that the 26S proteasome activity undergoes a functional modulation by IDE through an extra-catalytic mechanism. The IDE-26S proteasome interaction was analyzed during the Heat Shock Response and we report novel findings on IDE intracellular distribution that might be of critical relevance for cell metabolism.

## Introduction

Insulin-Degrading Enzyme (IDE) is an evolutionarily conserved 110-kDa zinc metallo-peptidase which belongs to the “Invertzincin” family (M16) of metallo-enzymes [1,2]. In mammalian tissues IDE expression is ubiquitous and the enzyme displays a wide sub-cellular distribution: it is found in the cytosol, endosomes, peroxisomes and a small fraction is further located on plasma membrane and mitochondria [3–7].

Several studies have shown that IDE efficiently degrades insulin, glucagon, beta-amyloid and, in general, peptides that share amyloidogenic propensity [8,9]. In this context, a role of IDE in dys-metabolic (*i*.*e*., type 2 Diabetes Mellitus) and neurodegenerative (*i*.*e*., Alzheimer’s Disease) disorders has been envisaged [10–13]. However, the biological properties of the enzyme raise *in vivo* some unresolved questions, since a pivotal role in insulin catabolism likely does not account for the relative abundance of IDE in tissues wherein glucose uptake is poorly affected by the hormone. Furthermore, it is still matter of debate whether the degradation of those natural substrates identified thus far [8–13] indeed reflects IDE main physiological role, since the enzyme is mainly located in cell compartments where the occurrence of anabolic and catabolic processes of these substrates is still matter of debate [14]. Hence, there is a compelling evidence that IDE could play additional roles not fully characterized yet. In this respect, it must be outlined that a plethora of intriguing observations (such as the widespread up-regulation of IDE in human tumors, the interaction with some onco-suppressors and with monomeric ubiquitin) demands deeper considerations on the interactome of IDE in the intracellular compartment [6,15–23]. As a matter of fact, we have recently published a paper whereby we proposed the enzyme as a novel Heat Shock-like Protein (HSP) [22], demonstrating that (*i*) in SHSY5Y cells IDE co-immunoprecipitates with the 26S proteasome and (*ii*) modulation of IDE expression influences the Ubiquitin-Proteasome System (UPS), affecting neuroblastoma cell proliferation and viability.

The 26S proteasome is the multi-subunit component of the Ubiquitin-Proteasome System which provides the machinery for the degradation of the vast majority of intracellular proteins, hence regulating the quality control and every aspect of cell life, as now widely recognized [24–28]. It is composed by one or two Regulatory Particles (RP), called 19S (MW ~900 kDa), which associate with a Core Particle (CP), named 20S (MW ~650 kDa), where the proteolytic activities reside [29,30]. From the structural viewpoint, the 19S RP is made by several subunits which self-assemble in the course of an ordered maturation process into two main complexes: the lid and the base [31]. On the other hand, the 20S CP has a cylindrical shape and it is composed by four-stacked rings with different specificities whose hierarchical assembly shapes a narrow channel through which the substrate is addressed for catalysis [32]. The two outer α-rings are made by seven structurally similar subunits, named alpha_1–7_, whose *N*-termini strictly regulate pore gating. The two inner rings are made by seven beta subunits, named beta_1–7_, where the active sites for the three identified catalytic activities are located [32–34]; more specifically, the caspase-like, the tryptic-like and the chymotryptic-like activities are associated with the beta_1_ (PSMB1_)_, beta_2_ (PSMB2) and beta_5_ (PSMB5)subunits, respectively [24]. Gate-opening and substrate selection are tightly regulated processes which occur through structural and conformational interactions between the 19S and the 20S particles [34]. The most studied activity of the 26S proteasome is that on poly-ubiquitinated substrates: the Ubiquitin-Conjugation System (UCS) selectively tags the target protein with a poly-ubiquitin chain of variable length which is then recognized by the RP. The detachment of ubiquitin moieties and denaturation of the protein precede its translocation into the proteasome catalytic chamber where the three activities generate peptides ranging from 3 to 20 residues [35,36]. Substrates undergoing proteolytic cleavage and fragmentation are not only Ub-tagged proteins, since also damaged and oxidized proteins can be degraded by the proteasome machinery regardless of the Ub-label [37,38].

It is worth pointing out that the emerging aspects on proteasome biology concern the co-existence of a highly heterogeneous population of proteasome particles that might be distinguished on the basis of their subunit composition as well as of their sub-cellular localization. Besides the 19S RP, alternative regulatory particles have been identified (*e*.*g*., PA28) and alternative catalytic subunits have been documented, being characterized by distinct substrate specificities and proteolytic properties (*e*.*g*., the immuno-proteasome) [39–42]. It is reasonable that additional not yet identified regulatory particles might exist. Their assembly with the CP could give rise to different complexes characterized by a precise biological function. Moreover, a myriad of molecular factors and a wide range of post-translational modifications modulate the assembly, the catalytic properties and the qualitative and quantitative range of substrates undergoing degradation by the proteasome [24,43–47].

In this paper, starting from the evidence that IDE and the 26S proteasome co-immunoprecipitate [22,48,49], we investigate such a tight interaction through cellular and biochemical approaches. Further, we provide the first characterization of the functional properties of the cytosolic pool of the 26S proteasome during 24 hr of recovery after heat stress exposure, formulating a proposal for the putative role of IDE in the observed behaviour. Notably, novel intriguing insights on IDE biological features during the HSR are discussed.

## Materials and Methods

### IDE-silencing experiments

Silencing of IDE expression in SHSY5Y cells was achieved through administration of a pool of Anti-Sense Nucleotide (siRNA) (Dharmacon Lafayette, St. Louis, MO, USA).

Differently from what reported in the previously published paper [22], the final concentration of the siRNA was adjusted to a sub-lethal concentration (*i*.*e*., 0.70 micromol/L instead of 1 micromol/L which was found to be lethal 72 h after the administration) that induced only a modest decrease of the proliferation rate without interfering with cell viability. In all experiments, Trypan blue exclusion viability test indicated that 96 h after anti-sense oligonucleotide administration at least 90% of cells was still viable.

Cells were seeded on a Falcon 6-well Plate at a concentration of 1.5 x 10^4^ cells/well in DMEM supplemented with 10% FBS. On the following day, the medium was removed and the cell monolayer gently washed twice with pre-warmed PBS before the addition of the transfection medium (Accell media, supplied by the manufacturer), containing either 0.70 micromol/L siRNA to IDE or 0.70 micromol/L of a non-targeting siRNA or else the equivalent volume of the siRNA vector (not specified by the manufacturer).

Cells were then grown in a humidified 5% CO_2_ incubator and harvested 48 h or 72 h after anti-sense administration. The cells were then lysed in detergent-free buffer (0.25 mol/L Sucrose, 0.025 mol/L Hepes, 0.01 mol/L MgCl_2_, 1 millimol/L EDTA, 1 millimol/L DTT, 2 millimol/L ATP, pH 7.8) through freeze-thaw cycles for preparation of crude cell extracts [33,46,50]. The soluble fraction containing the 26S proteasome was then “squeezed out” through centrifugation 30 min at 14,000 rpm at 4°C. Time-course analysis of the 26S proteasome activity during 24 h of heat-stress recovery was performed by exposing cells for 20 min at 46°C in a thermostated incubator; then, recovery phases occurred under standard conditions (37°C, 5% CO_2_). It is worth pointing out that heat stress procedure was made cultivating cells both in Transwell 6-well Plates and standard cell flasks: the temperature increase of cell culture medium was monitored and, notably, during the 20 minutes time interval the cell medium raised the effective 46°C temperature around the 16^th^ minute, thus determining an overall exposure of cells to 46°C for 4 minutes.

At the indicated different time-points, *i*.*e*. before heat-stress was administered (corresponding to time = 0) and after 2 h, 6 h, 12 h and 24 h of recovery cells were harvested as indicated above. In all cases Trypan blue exclusion viability test indicated that, after stress, at least the 85% of cells was viable over 24 h of recovery.

It must be remarked that in this study SHSY5Y cells were silenced in an insulin-free medium (according to the manufacturer instructions) and, further, no supplementation with FBS was made; this procedure was followed to avoid the presence of insulin which was reported to affect the 26S proteasome activities [48,49].

### Proteasome assay in SHSY5Y cells

Proteasome assay was performed in crude cell extracts from SHSY5Y cells in a 96-well microplate for fluorimetric readings. The fluorescence was detected in an Eclipse fluorimeter (Varian) and the excitation and emission wavelengths were, for all substrates employed, 380 nm and 460 nm, respectively.

Protein concentration in crude cell extracts was normalized by Bradford assay and (for each experimental condition) 5 micrograms were incubated in the 26S proteasome activity buffer (0.05 millimol/L Hepes, pH 7.8, 20 millimol/L KCl, 5 millimol/L MgCl_2_, 1 millimol/L DTT, 1 millimol/L ATP) with 200 micromol/L of the three different fluorogenic substrates specific for the 26S proteasome proteolytic activities (*i*.*e*., Suc-LLVY-AMC, Boc-LRR-AMC and Z-LLE-AMC for the chymotryptic-like, tryptic-like and caspase-like activities, respectively) (Boston Biochem, Boston, USA). Each measurement was done in triplicate and the fluorimetric reports were then collected every 30 min over 4 h of incubation at 37°C. All buffers contained Mg^2+^ and ATP to improve stability of the 26S complex and KCl to reduce the activity of the 20S proteasome pool [47,50].

All the results here reported represent the average data from three different observations on five independent experiments: each measurement and the relative activities (expressed as *a*.*u*.) reflect the rate of fluorogenic substrate hydrolysis by the 26S proteasome with an extent of peptide hydrolysis less than 10%. Each measurement was further performed in presence of 10 micromol/L (for chymotryptic-like and caspase-like activities) and 50 micromol/L (for tryptic-like activity) lactacystin in order to distinguish the specific 26S proteasome proteolytic activity from not specific enzymatic activity. Noteworthy, not specific degradation of the fluorogenic substrates was essentially negligible for chymotryptic and caspase-like activities, whereas only a modest tryptic activity not referable to the 26S proteasome was detected (around 15–20% of the total activity).

### Kinetic analysis

The characterization of the 26S proteasome activities (*i*.*e*. chymotryptic-like, tryptic-like and caspase-like) as a function of IDE concentration was carried out through a fluorimetric approach. All measurements were performed in quartz fluorimeter cuvette.

A highly purified 26S proteasome, extracted from transformed HEK cells (Boston Biochem, Boston, USA) was diluted to a final concentration of 1 nanomol/L in the assay buffer (Tris-HCl 20 millimol/L, 10 millimol/L MgCl_2_, 10% glycerol, 2 millimol/L DTT, 1 millimol/L ATP, pH 7.8) in the absence and in the presence of different concentrations of a recombinant IDE expressed in *Spodoptera frugiperda* (Calbiochem, Merck Biosciences, Darmstadt Germany). The reaction mixtures were incubated 20 min at 37°C. Then, the fluorogenic substrate (50 micromol/L) specific for each of the three 26S proteasome activities was added. The rate of hydrolysis of the fluorogenic substrate was monitored for 45 min, a time interval over which only a small fraction of the substrate underwent proteolysis, and the relative velocities were extrapolated; during the same time interval, no auto-proteolysis of the substrate was observed.

The relative activities reported throughout the text refer to the ratio between velocities of the reaction in the absence and in the presence of IDE. The reaction was blocked upon administration of proteasome specific inhibitors (*i*.*e*., 20 micromol/L lactacystin and/or epoxomycin).

Kinetics of Z-LLE-AMC degradation by IDE were determined as follows: the fluorogenic substrate was incubated in the presence of 30 nanomol/L IDE in the assay buffer (Tris-HCl 20 millimol/L, 10 millimol/L MgCl_2_, 10% glycerol, 2 millimol/L DTT, 1 millimol/L ATP, pH 7.8) and the rate of hydrolysis was monitored over 45 min. No enzymatic activity by IDE was detected on chymotryptic-like (*i*.*e*., Suc-LLVY-AMC) and tryptic-like (*i*.*e*., Boc-LRR-AMC) substrates (Figs A and B in S1 Fig). On the other hand, thr enzymatic activity by IDE on the caspase-like (*i*.*e*., Z-LLE-AMC) substrate (Fig C in S1 Fig) was measured, allowing to obtain the catalytic parameters according to the following equation:

$$\frac{\left[E_{0}\right]}{v}=\frac{K_{m}}{k_{cat}}\cdot \frac{1}{\left[S\right]}+\frac{1}{k_{cat}}$$

where [*E_0_*] is the enzyme concentration, *ν* is the observed velocity and [S] is the substrate concentration. The resulting catalytic parameter are *K_m_*, corresponding to the apparent affinity constant (or Michaelis-Menten constant) of substrate for the free enzyme (to form the ES complex), and *k_cat_*, corresponding to the velocity of the rate-limiting step during the enzymatic activity.

### Western blotting analysis

Semi-quantitative analyses of IDE (Covance, Princeton, NJ, USA) and of the 26S proteasome, HSP70, GAPDH and poly-ubiquitinated proteins (Abcam, Cambridge, UK) were performed by Western blotting. The antibody to the 26S proteasome recognizes the p27 subunit of the complex.

20 μg of proteins from crude cell extract or whole cell lysates were separated on a 10% acrylamide gel under reducing and denaturing conditions. Separated proteins were then transferred to a HyBond-ECL nitrocellulose filters (Amersham Biosciences, Piscataway, NJ, USA) for 1 h at 4°C. Unsaturated binding sites were blocked by incubating filters in a 0.01% Tween-PBS, 5% fatty free milk solution. Filters were then probed with the specific antibody at the recommended concentration and, thereafter, incubated with a Horseradish Peroxidase-conjugated anti-rabbit or anti-mouse IgG antibody (Biorad, Hercules, CA, USA), diluted 1:50000 in 0.2% Tween-PBS fat-free milk. Immunoreactive signals were detected with an ECL Advance Western Blotting Detection Kit (Amersham Biosciences).

### Immunofluorescence and Confocal Microscopy

SHSY5Y cells were grown in high-glucose DMEM supplemented with 10% FBS, L-glutammine (2 mM), penicillin (50 IU/ml), streptomycin (50 microg/ml), sodium pyruvate (1 millimol/L). 24 h after heat-stress administration (46°C, 20 min), control (not-stressed) and stressed SHSY5Y cells were rinsed in phosphate buffered saline (PBS) and directly fixed in 4% (w/v) paraformaldehyde for 15 min at room temperature. The fixed cells were blocked and permeabilized with 3% (w/v) bovine serum albumin and 0.2% (v/v) Triton X-100 in PBS (blocking solution) for 20 min. For immunostaining, cells were incubated for 1 h with an anti-IDE rabbit polyclonal antibody (Covance, Princeton, NJ, USA) together with either the anti-P26S or anti-Na^+^/K^+^ATPase mouse monoclonal antibodies (Abcam, Cambridge, UK). Slides were also incubated with an anti-IDE mouse monoclonal antibody (Covance, Princeton, NJ, USA), together with either anti-Giantin or anti-calnexin rabbit polyclonal antibodies (respectively, Golgi and ER Markers, Abcam, Cambridge, UK). After rinsing in PBS, cells were incubated with secondary antibodies, anti-rabbit conjugated with fluorescein isothiocyanate (FITC) (Vector Laboratories, USA) or conjugated with Alexa Fluor-633 (Invitrogen, USA) and anti-mouse conjugated with Rhodamine Red or FITC (Invitrogen, USA), diluted in blocking solution for 1 h at room temperature. Cells were washed with PBS and nuclei counterstained with DAPI (Sigma-Aldrich St Louis, CO). Images (1024x1024 pixel) of the cells were acquired sequentially using a confocal laser scanning system (TCS-SP2, LeicaMicro-systems, GmbH, Wetzlar, Germany, 63x/1.4 oil-immersion objective) with identical settings for laser power, gain and offset. Images were analyzed with ImageJ program (http://rsbweb.nih.gov/ij/) and composed using Photoshop software (Adobe System, USA). Laser excitation at 488 nm of the sample was followed by an excitation at 543 nm or 633 nm to collect emission signals from FITC and rhodamine or Alexa Fluor-633, respectively. DAPI staining was imaged by two-photon excitation (740 nm, < 140 fs, 90 MHz) performed with an ultrafast, tunable, mode-locked Ti: Sapphire laser (Chameleon, Coherent Inc., Santa Clara, CA).

### Co-Immunoprecipitation

An anti-IDE monoclonal antibody or not-specific mouse IgG, as internal control (Abcam, Cambridge, UK) were coupled to M-270 epoxy Dynabeads (Invitrogen) as indicated by the manufacturer (5 micrograms of Ab/1,5 milligrams of beads). Magnetic beads coated with anti-IDE Ab were stored at 4°C in PBS, 0.02% NaN3. Before use, coated beads were washed three times with lysis buffer. SHSY5Y cells were cultivated under standard conditions and heat-stressed 20 min at 46°C. Total protein extracts were obtained by rinsing the cells twice with ice-cold PBS followed by the addition of ice-cold lysis buffer (Co-IP), supplemented with 1 millimol/L DTT, 50 millimol/L NaCl and a protease-inhibitor cocktail with broad specificity (Sigma-Aldrich St Louis, CO). Harvested cells were washed once in PBS and the pellet was resuspended in the Extraction Buffer (cell mass to Extraction Buffer ratio 1:9) supplemented with a protease inhibitor cocktail, according to manufacturer instruction (Invitrogen). Cells were then incubated for 15 min in ice and centrifuged at 2600 x g for 5 min, 4°C. The extract was used immediately for co-immunoprecipitation. Purification was achieved by slow mixing at 4°C. The isolated protein complex was eluted from the beads for 20 min at room temperature in a fresh aqueous solution.

### Subcellular Fractionation

Isolation of Endoplasmic Reticulum fraction from resting and heat-exposed SHSY5Y cells was performed by following the method described elsewhere [51].

Briefly, SHSY5Y cells were grown under standard aerated conditions, heat-stressed and recovered for 24 h. Afterwards, cells were harvested, resuspended in an isotonic buffer (0.27 mol/L mannitol, 0.01 mol/L Tris-base, 100 millimol/L EDTA, 1 millimol/L PMSF, pH 7.4) and lysed through sonication. Cell lysates were then centrifuged 10 min at 1400 g, 4°C. A small aliquot of the supernatant was then collected and labeled as total cell lysate.

The remaining supernatant was further centrifuged 10 min at 150,000 g at 4°C; the resulting brown pellet (crude mitochondria) was then discarded. The crude ER (supernatant) was then loaded on a sucrose gradient and centrifuged for 70 min at 152,000 g in an SW41 rotor at 4°C. A small aliquot of the upper solution was then collected and labeled as cytosol, whereas the white and dense band at the sucrose interface, representing the ER fraction, was carefully withdrawn and diluted in the previously described isotonic buffer.

An additional centrifugation 45 min at 126,000 g at 4°C was performed to further purify the ER fraction. The pellet was then resuspended in PBS 1x. Samples were then normalized through Bradford assay and stored at -80°C until use. A mouse-polyclonal anti-calnexin antibody (Abcam, Cambridge, UK) was used to stain the ER fraction.

### Limited tryptic digestion of ER fraction

The ER fraction purified either from resting cells or recovered cells (24 h of recovery) were exposed to limited proteolytic digestion by 0.05% trypsin, supplemented with 1 millimol/L EDTA, for 30 min at 30°C as described elsewhere [52]. As an internal control, samples were incubated over the same time interval in the presence of 1 millimol/L EDTA and in the absence of trypsin. Reaction was then blocked by adding standard sample buffer for electrophoresis and heat-denatured. Thereafter, the samples were run on SDS-PAGE and blotted to a nitrocellulose membrane for Western blotting analysis. Filters were then probed with a polyclonal anti-IDE antibody, an anti–calnexin (ER Marker) (Abcam, Cambridge, UK) and an anti-Erp57 (Santa-Cruz, USA) rabbit polyclonal antibodies.

### Purification of proteasome particles

Proteasome particles from the cytosolic fraction of SHSY5Y cells were affinity purified by using a kit according to manufacturer instructions (Merck Millipore, Germany).

To improve proteasome stability along the purification procedure, the cytosolic fraction was supplemented with 10% glycerol. Purified particles were then denatured and analyzed by Western blotting to get a semi-quantitative information on IDE association with 26S proteasome under resting and heat-stress conditions.

It is worth pointing out that to maximize reliability of the experimental outcome, equal amounts of cytosolic extract from the two experimental conditions were suspended with the affinity beads. Furthermore, the purified proteasome pools were normalized by Bradford assay.

### Statistical analysis

One-way analysis of variance (ANOVA) was used to assess statistically significant differences among groups and Tukey’s honestly significant difference post hoc test was used for pairwise comparisons after the analysis of variance.

## Results

### Proteasome activity in IDE-silenced neuroblastoma cells

We previously showed that IDE behaves as an Heat Shock Protein and that it co-purifies with the 26S proteasome [22]. Further we demonstrated that, in SHSY5Y neuroblastoma cells, extensive down-regulation of IDE expression (by administering 1 micromol/L IDE-targeting siRNA) triggers apoptosis in association with a significant decrease in poly-ubiquitinated (poly-Ub) proteins content [22].

**Fig 1:**
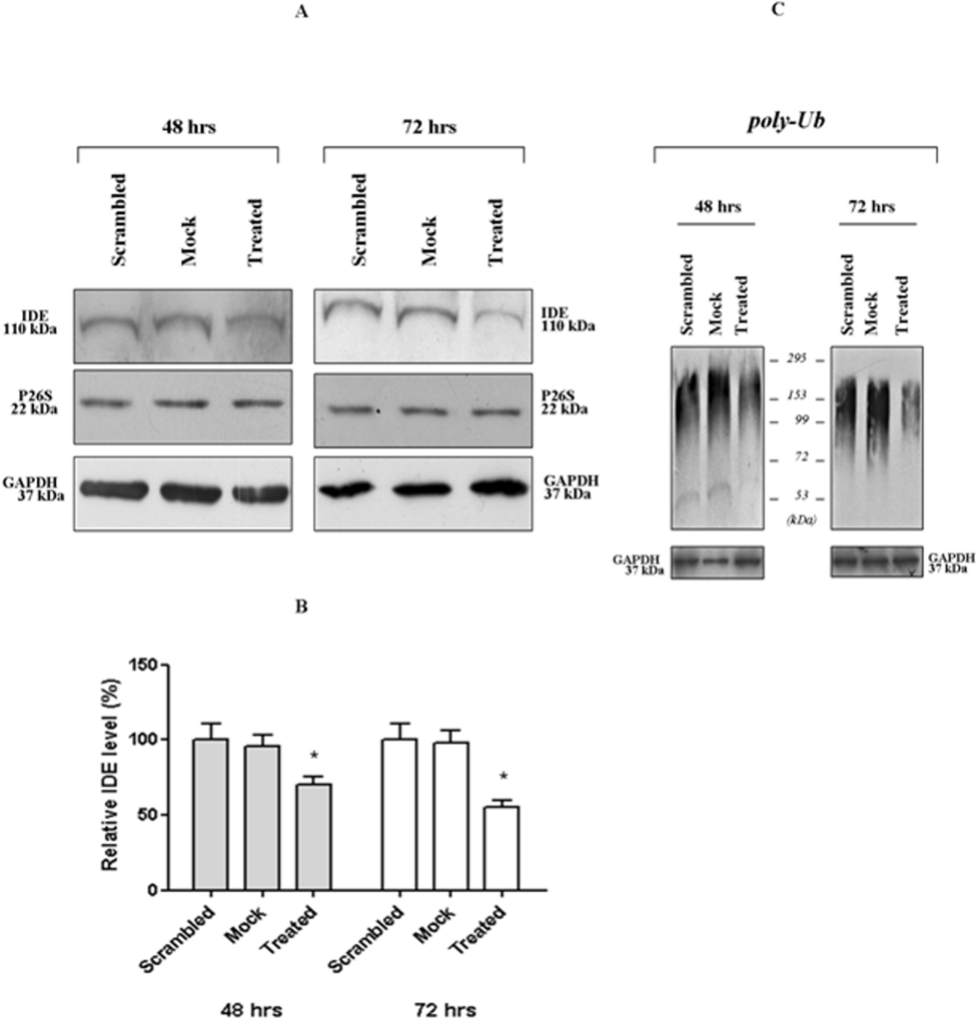
Western blotting analysis of crude cell extracts from treated (IDE-silenced) and *wild-type* (scrambled and mock) SHSY5Y. Cell extracts were harvested 48 h and 72 h after the administration of 0.7 mircomol/L anti-sense oligonucleotides and analyzed through Western blotting. Filters were probed with antibodies specific for IDE (110 kDa), P26S (22 kDa), GAPDH (37 kDa) and (C) poly-ubiquitinated proteins. A representative immunoblot of five independent experiments is shown. (B) Densitometric analysis of IDE signals from the Western blotting is shown in Fig 1A.

**Fig 2:**
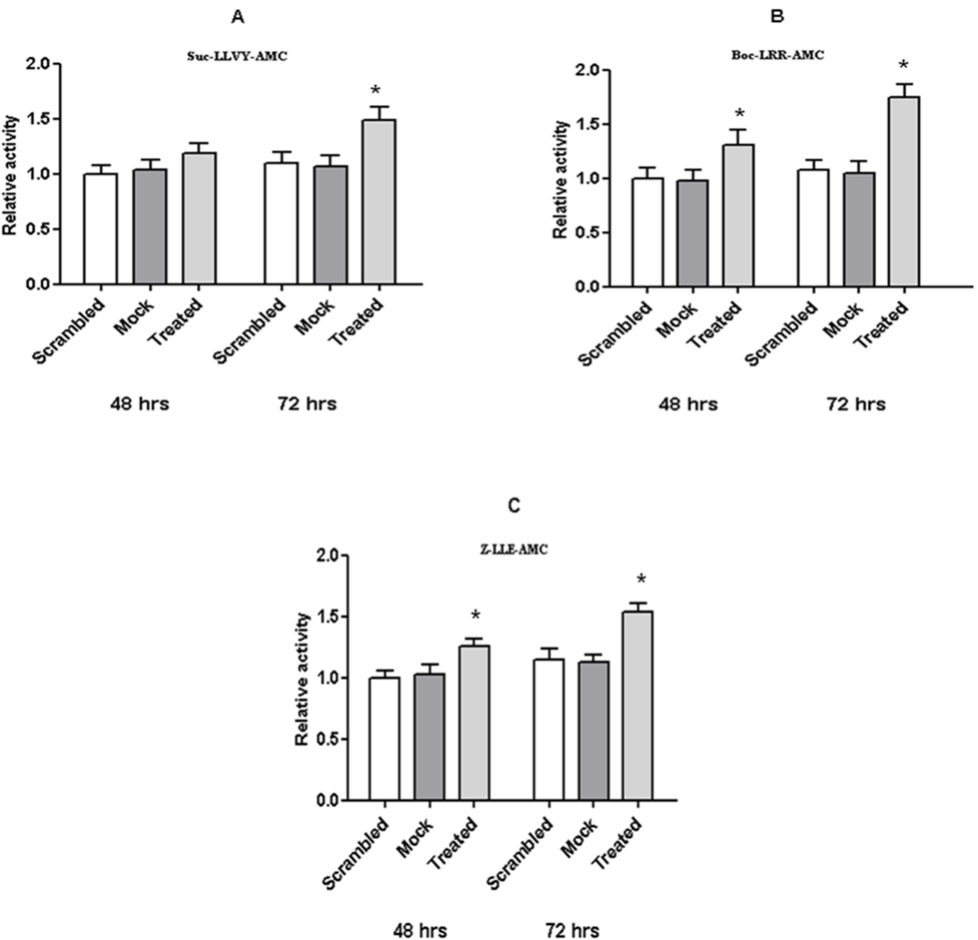
26S proteasome activity in crude cell extracts from treated and *wild-type* SHSY5Y. The proteasome assay on crude cell extracts from either treated or *wild-type* cells was performed 48 h and 72 h after the anti-sense oligonucleotide delivery. The chymotryptic-like (A), the tryptic-like (B) and the caspase-like (C) activities of the 26S proteasome particles were assayed on specific fluorogenic substrates. Values reported are the means +/- S.E. of five independent experiments. *, significantly different from control (*p*<0.05, one way ANOVA, followed by Tukey’s test, *n* = 15).

In the present study, we first investigated the effect of a sub-lethal IDE siRNA dose (so as to decrease IDE levels but avoiding apoptosis) on the modulation of the 26S proteasome activity and poly-Ub proteins turnover. To this purpose, 0.7 micromol/L IDE-targeting siRNA (treated) was administered to SHSY5Y cells and their viability monitored over 96 h time period after treatment; only a minimal decrease in the proliferation rate was detected (see Materials and Methods) without a decrease in cell viability, which was always higher than 95%. As an internal control, SHSY5Y cells were treated either with the siRNA vector alone (scrambled) or else with a not-targeting pool of siRNA (mock). Notably, results from scrambled and mock cells were fully comparable, as indicated in Figs 1 and 2: therefore, they will be not discussed separately throughout the text below and they will be referred as *wild-type* cells.

Then, crude cell extracts (*i*.*e*. the soluble fraction of cells, obtained upon detergent-free lysis procedure) [34,47,50] from each experimental condition were harvested 48 and 72 h after anti-sense oligonucleotide delivery and investigations carried out through Western blotting indicated that: *(i)* a limited reduction of intracellular IDE concentration in treated cells was effectively obtained (*i*.*e*. 30% at 48 h and 50% at 72 h) (Fig 1A and 1B); *(ii)* no detectable variation in the overall content of 26S proteasome and GAPDH both in treated and *wild-type* cells was occurring (Fig 1A).

Hence, a Western blotting analysis was set up to monitor the content in poly-ubiquitinated proteins in the extracts. Fig 1C clearly indicates that, after 72 h from IDE-silencing, the limited down-regulation of IDE expression in treated cells (*i*.*e*., 50%) (Fig 1A and 1B) was enough to get a significant reduction in poly-ubiquitinated proteins content in treated cells. The effect was even less pronounced after 48 h from IDE-silencing (Fig 1C). Therefore, we concluded that the phenomenon occurred regardless of apoptotic stimuli induction, suggesting a critical contribution of IDE decrease.

Thereafter, to further investigate whether the observed behaviour depended on the increased turn-over of poly-ubiquitinated proteins rather than the impaired synthesis, the three enzymatic activities of the 26S proteasome were assayed employing the Suc-LLVY-MCA, Boc-LRR-AMC and Z-LLE-AMC fluorogenic substrates, which are considered to be specific for the chymotrpytic-like, tryptic-like and caspase-like activities, respectively. Interestingly, the hydrolysis of 200 micromol/L of the three substrates by crude cell extracts from treated cells displayed kinetics faster than that observed by extracts from *wild-type* cells (Fig 2). Further, the behaviour seemed to be referable to the rate of IDE depletion, being maximally evident in samples harvested 72 h after siRNA delivery (Fig 2). In details, the kinetics of Suc-LLVY-AMC hydrolysis (referable to the chymotryptic-like activity of 26S proteasome) showed a 15% and 50% increase after 48 h and 72 h of silencing, respectively (Fig 2A) in treated cells with respect to *wild-type* cells. Similarly, the kinetics of Boc-LLR-AMC hydrolysis (referable to the tryptic-like activity of 26S proteasome) were increased by 30% and 60% at 48 and 72 h, respectively (Fig 2B), and the kinetics of Z-LLE-AMC (referable to the caspase-like activity of 26S proteasome) were increased by 25% and 40% at 48 and 72 h, respectively (Fig 2C).

### Enzymatic Characterization of IDE-26S proteasome Interaction

These results raised the question whether the up-regulation of the 26S proteasome activity in IDE-silenced SHSY5Y occurred through mechanisms dependent on a direct IDE interaction or, instead, through unknown pathways indirectly linked to IDE-depletion.

**Fig 3:**
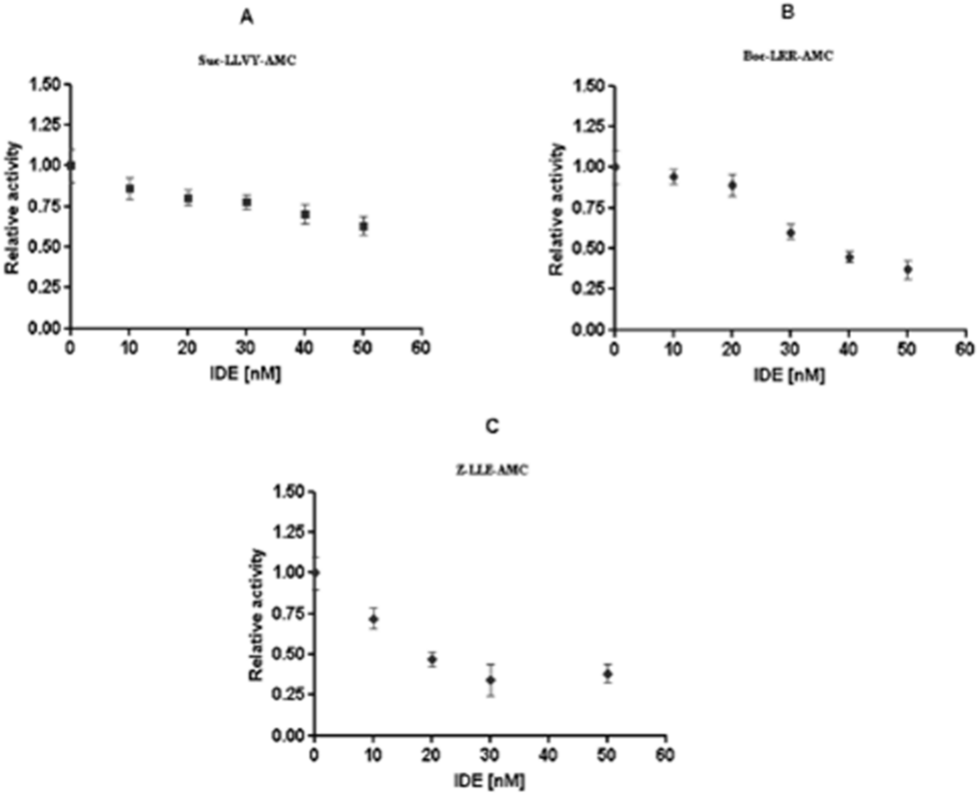
Biochemical characterization of IDE-26S proteasome interaction. The chymotriptic-like (A), tryptic-like (B) and caspase-like (C) activities of a highly purified 26S proteasome were assayed on specific fluorogenic substrates in presence of highly purified eukaryotic IDE. The reported values represent the ratio between the velocity of degradation of 50 micromol/L fluorogenic substrate by 1 nM 26S in the absence and the presence of different IDE concentrations, ranging from 10 nanomol/L to 50 nanomol/L. Results presented are the means +/- S.E. of five independent experiments.

To address this issue, and to get a mechanistic insight on IDE effect on the proteasome activity, a biochemical study by using a highly purified preparations of 26S proteasome and eukaryotic IDE was carried out. The kinetics of degradation of 50 micromol/L fluorogenic substrates (*i*.*e*., Suc-LLVY-AMC, Boc-LLRAMC and Z-LLE-AMC for the chymotryptic, tryptic and caspase-like activities, respectively) by 1 nanomol/L 26S proteasome, in standard activity buffer at 37°C, in the presence of IDE concentrations ranging from 1 nanomol/L to 50 nanomol/L are reported in Fig 3.

Through these fluorimetric analyses, a consistent dose-dependent inhibition of the three enzymatic activities of the 26S proteasome in the presence of IDE was observed, at least over the investigated concentration range. In detail, in the presence of 50 nanomol/L IDE, the kinetics of Suc-LLVY-AMC degradation was slowed down by 55% (Fig 3A), whereas, the kinetics of Boc-LLR-AMC cleavage was reduced by 65–70% (Fig 3B); the reported values were referred to the activity observed for the 26S proteasome alone under the same experimental conditions. It must be outlined that the activity observed was highly specific for the 26S proteasome, since the reaction was blocked exclusively upon administration of proteasome specific inhibitors (*i*.*e*., 20 μM lactacystin and/or epoxomycin) and IDE alone did not show any enzymatic activity on the Suc-LLVY-AMC and Boc-LLR-AMC fluorogenic substrates (S1A and S1B Fig).

Conversely, in the case of the caspase-like activity some additional considerations are required, sinceIDE was found to be able to cleave the fluorogenic Z-LLE-AMC substrate *in vitro* under this experimental condition (calculated *k*_cat_/K_M_ = 1.93 x 10^2^ M^-1^s^-1^) (S1C Fig). Therefore, the caspase-like activity specific for the 26S proteasome was derived by subtracting the enzymatic kinetics (moles/min) obtained from IDE alone at various concentrations, from the catalytic activity of the 26S proteasome observed in the presence of the same IDE concentrations. The relative 26S proteasome activity dependence on IDE concentration, reported in Fig 3C, corresponds to that obtained after this normalization and it clearly indicates that kinetics of Z-LLE-AMC degradation by 1 nanomol/L 26S proteasome underwent 60% inhibition in the presence of 50 nanomol/L IDE.

Furthemore, it is noteworthy that the modulation of the 26S proteasome activity by IDE, reported in Fig 3A and 3C, could be also obtained when IDE had been previously incubated with a Zn^2+^ chelator (*i*.*e*., EDTA), which inhibits IDE enzymatic activity for its fluorogenic substrate (S2 Fig). This result indeed suggests that also enzymatically inactive IDE is able to affect the 26S proteasome activity.

### Time-Course of 26S proteasome activity over 24 h of recovery after heat-stress in IDE-silenced and wild-type cells

It is widely recognized that the UPS plays a critical role during the Heat Shock Response, even though the modulation of the 26S proteasome activity and the expression of the different subunits greatlyvaries depending on the type and the intensity of the stress and also on the cell model investigated, likely reflecting the crowdness of UPS modulator in the intracellular compartment [36,53–56]. In this respect, the evidence that IDE metabolism is affected by stress and the above described effect on the 26S proteasome activity envisaged a tight contribution of the IDE-26S proteasome interaction to the HSR.

Since some authors previously described some physiological situation wherefore the IDE-26S proteasome interaction was lost [48,49], we first checked whether in heat-stressed SHSY5Y cells the interaction still occurred. Thus, confocal microscopy and co-immunoprecipitation assay on resting and heat-stressed SHSY5Y demonstrated that IDE and the 26S proteasome diffusely co-localized and co-immunoprecipitated in both experimental conditions (S3 Fig)

Therefore, a comparison between the activity of proteasome particles from *wild-type* and IDE-silenced SHSY5Y cells during the HSR was performed. Noteworthy, IDE-silencing was carried out by following the non-lethal conditions indicated above, whereas the heat-stress (*i*.*e*., 46°C for 20 min) was administered 48 h after siRNA delivery. Since the 26S proteasome activity from scrambled and mock cells was fully comparable, even in this case, these two experimental conditions will not be discussed separately throughout the following text and they will be referred to as *wild-type* cells.

**Fig 4:**
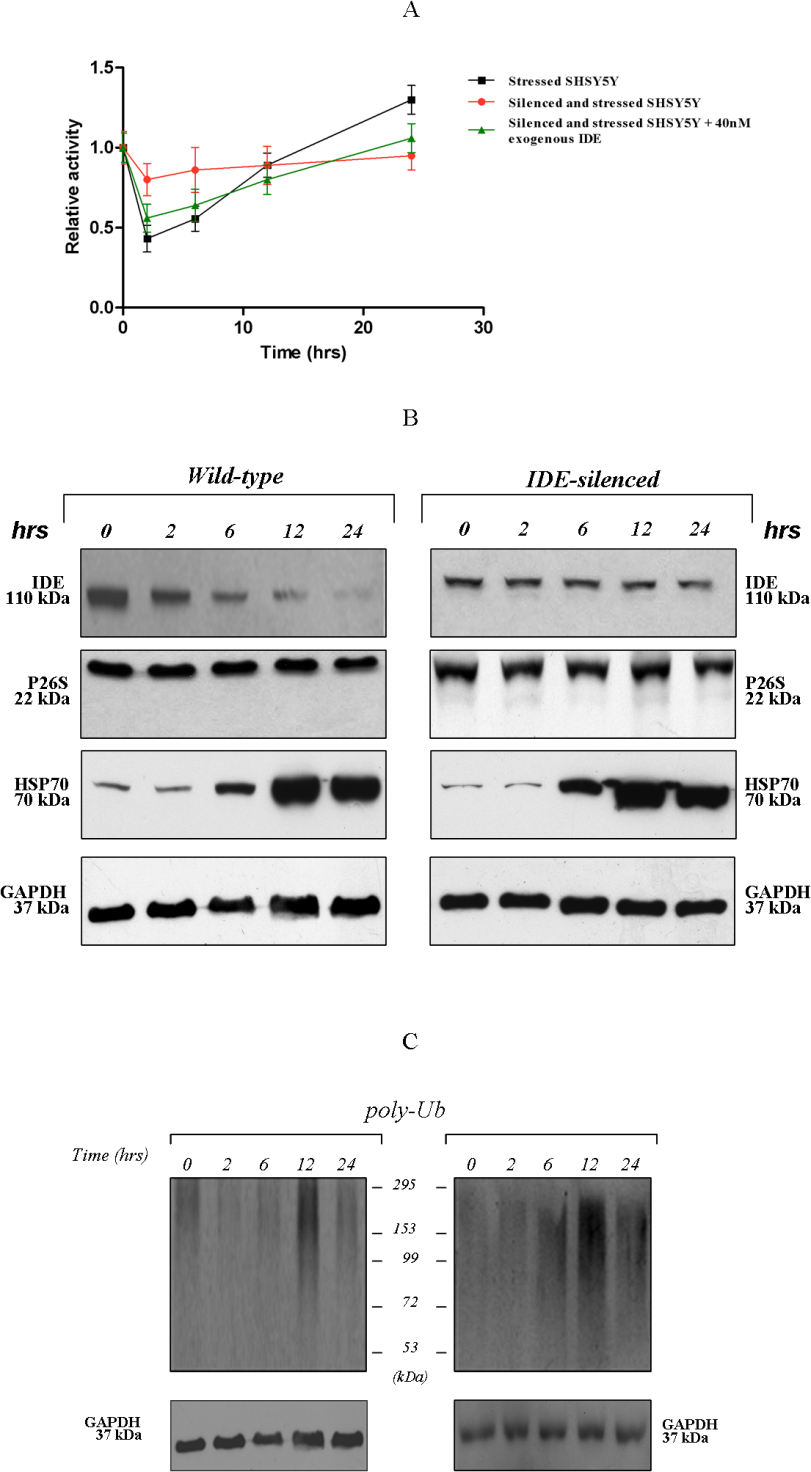
Time-course of 26S proteasome activity over the HSR and Western blotting analyses. (A) The chymotryptic-like activity of 26S proteasome particles partially purified from *wild-type* (black square), treated (red circle) and in presence of 10–40 nanomol/L exogenous IDE added to extracts from treated cells (green triangle), was assayed through the proteasome assay: the relative activity represents the ratio between the velocity of degradation of 200 micromol/L fluorogenic substrate at time 0 (resting conditions) *vs* the indicated time of recovery. The reaction was followed over 3 h of incubation at 37°C. Results presented are the means +/- S.E. of five independent experiments* (*p*<0.05, one way ANOVA, followed by Tukey’s test, *n* = 15). Western blotting analysis of crude cell extracts: (B) Filters were probed with antibodies specific for: IDE (110 kDa), 26S proteasome (22 kDa), HSP70 (70 kDa), GAPDH (37 kDa). (C) Filters were probed with a polyclonal antibody specific for ubiquitin. A representative immunoblot of five independent experiments is shown.

Kinetics of Suc-LLVY-AMC degradation by 26S proteasome particles from *wild-type* cells dramatically varied over the recovery phases: at time 0 of heat-stress recovery, the kinetics of substrate cleavage was fully comparable to that of untreated cells (not-stressed). However, within the first two hours, the 26S proteasome activity underwent progressive and consistent down-regulation, by reaching about 45% (SD ± 5%) of the original activity (Fig 4A). Thereafter, the activity slowly recovered, being, instead, consistently enhanced after 24 h of recovery with respect to 26S proteasome particles extracted from untreated cells grown over the same time-lapse under standard conditions.

Interestingly, such a pattern of 26S proteasome activity was lost in samples from IDE-silenced SHSY5Y (Fig 4A). The kinetics of Suc-LLVY-AMC cleavage, recorded at various time-points during the stress recovery, were substantially unaffected: thus, Fig 4A highlights the “flattened” profile of the proteasome activity behaviour in IDE-silenced SHSY5Y cells, even though a minimal decrease during the first phase was observed. Notably, the exogenous addition of tailored concentrations of eukaryotic IDE (10–40 nanomol/L) to the crude cell extracts from IDE-silenced cells before the administration of the fluorogenic substrate were sufficient to restore the original pattern of the activity during the HSR (Fig 4A). This approach was accompanied, as an internal control, by a semi-quantitative analysis of the 26S proteasome, HSP-70 and GAPDH contents in the crude cell extracts (Fig 4B), and no significant variation between *wild-type* and IDE-silenced cells was observed. Thus, the relative abundance of the 26S proteasome and GAPDH was unaffected during the 24 h of recovery, whereas the HSP70 was markedly induced. Then, poly-ubiquitinated proteins content was analyzed and, even in this case, no variations between *wild-type* and IDE-silenced cells was observed (Fig 4C). In detail, even though in IDE-silenced cells a reduced basal content under resting conditions (*i*.*e*. time 0) was confirmed to occur, consistently with our previous publication and with Fig 1C [22], a dramatic decrease of the poly-Ub proteins was detected during the first 6 h of recovery for both *wild-type* and IDE-silenced cells (Fig 4C). At later times (12 h) a significant accumulation in poly-Ub was then documented for both *wild-type* and IDE-silenced cells, followed by a recovery of its original content (after 24 h) (Fig 4C).

### IDE distribution in the cell during the HSR

**Fig 5:**
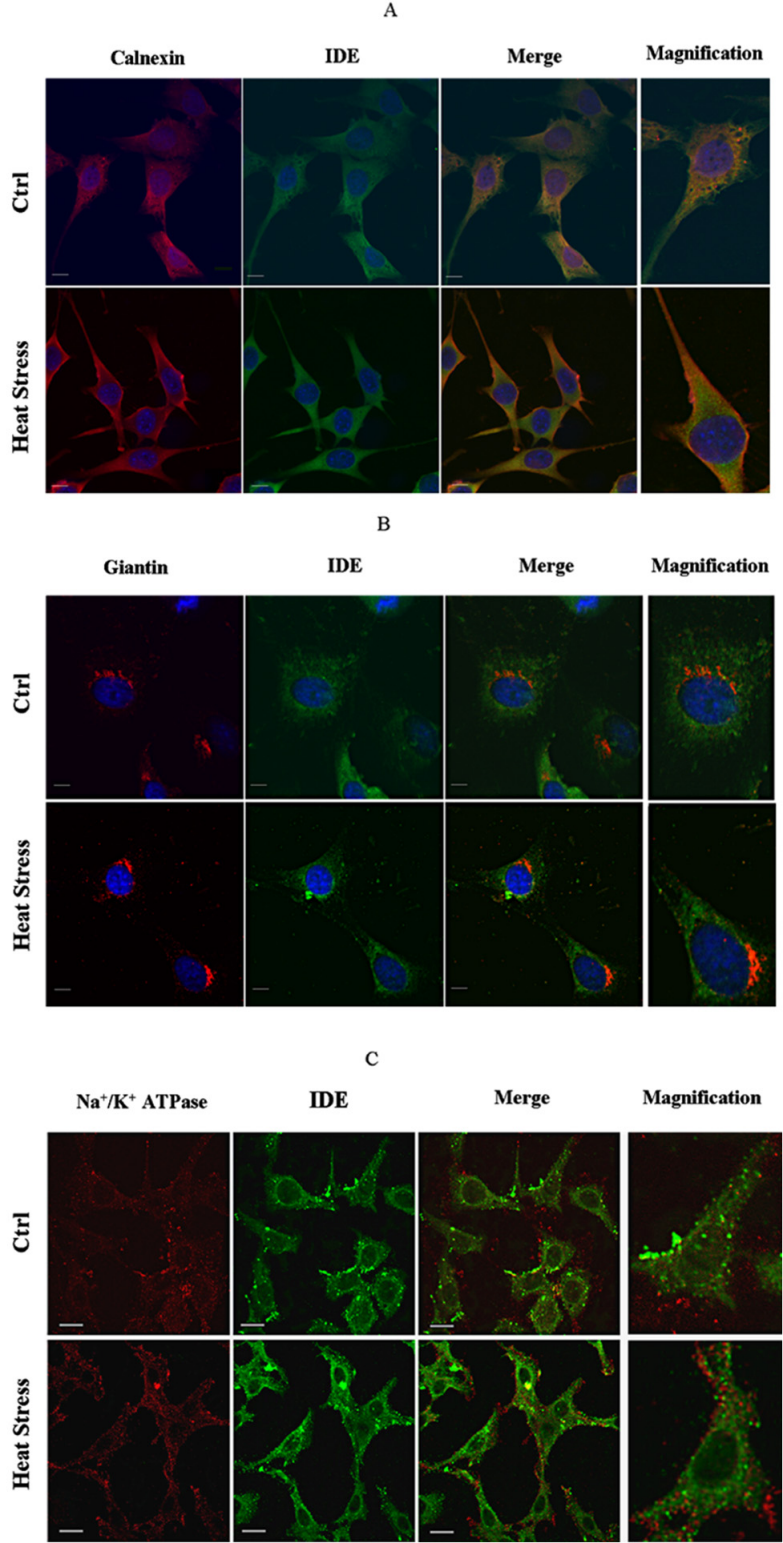
Confocal images of control and stressed fixed SHSY5Y cells 24 h after heat administration. Cell localization of IDE was determined using anti-IDE mouse monoclonal (A and B) or anti-IDE rabbit polyclonal (C) antibodies. The Endoplasmic Reticulum was stained with anti-calnexin (A), while Golgi was labelled with anti-giantin rabbit polyclonal antibody (B). Plasma membrane was stained with anti-Na^+^/ K^+^-ATPase mouse monoclonal antibody (C) Nuclei were counterstained with DAPI (blue)(A,B). Bars correspond to 20 micrometers.

In our previous paper we demonstrated that IDE expression increased in whole cell lysate upon stress exposure [22]; herewith, we focused instead our attention on the same crude cell extracts (*i*.*e*. the soluble fraction of cells, obtained upon detergent-free lysis procedure) where measurements of the 26S proteasome activity were undertaken. Through semi-quantitative analysis by Western blotting, we observed the occurrence of a significant and progressive reduction in IDE content in the cytosol under stressful conditions, being maximal after 24 h of recovery (Fig 4B). Nonetheless, the observed behaviour was markedly less pronounced in IDE-silenced cells. At this stage, it is not possible yet to provide a reliable and exhaustive interpretation of this behaviour. Indeed, data on *wild-type* cells, indeed, clearly indicated that IDE content in the cytosol underwent a decrease during the HSR, in spite of an enhancement of the overall IDE cellular content; this observation suggested a translocation of IDE from the cytosolic fraction to some other cell compartments which were excluded from crude cell extract preparation. This suggestion was further reinforced by the evidence that IDE was accumulated in the membrane-enriched cell pellets which were discarded during crude cell extracts preparation (S4 Fig). In order to confirm that the reduced cytosolic content of IDE in heat-stressed SHSY5Y was mirrored by IDE accumulation in specific cell compartments, an immunofluorescence labelling of IDE in stressed and control *wild-type* SHSY5Y cells 24 h after heat administration was carried out, by using different subcellular markers. In particular, (*a*) calnexin was used to stain the endoplasmic reticulum (ER), (*b*) the anti-giantin antibody was employed to identify components of the *cis* and medial Golgi system, (*c*) the Na^+^/K^+^ ATPase to stain the cell membrane and (*d*) DAPI to label the nucleus. Interestingly, IDE redistribution occurred during the HSR from the cytosol toward the ER with ER signals markedly increasing in heat-stressed cells (Fig 5A). On the other hand, no localization of IDE in the Golgi was observed neither in control nor in stressed SHSY5Y cells (Fig 5B); further, under our control and stressful conditions, no translocation of IDE was detected neither toward the plasma membrane (Fig 5C) nor to the nucleus (Fig 5A and 5B).

**Fig 6:**
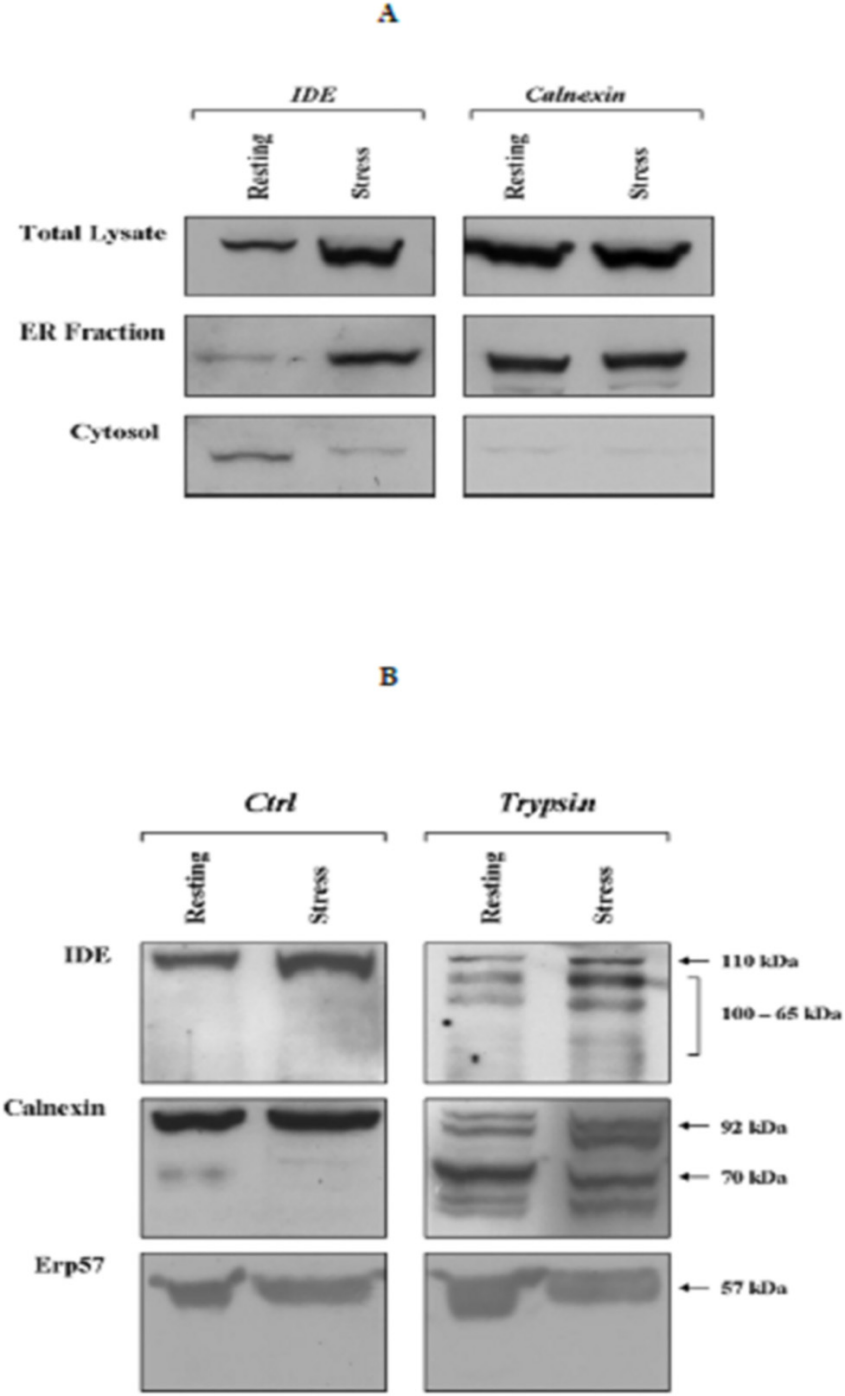
Analysis of IDE content in sub-cellular fractions and limited tryptic digestion of ER fractions. (A) Total lysates, Endoplasmic Reticulum fraction and cytosolic fraction were separated from resting (referred to as Ctrl) and stressed (referred to as Stress) SHSY5Y cells 24 h after heat administration and assayed by Western blotting. Filters were probed either with a anti-IDE (110 kDa) or a anti-calnexin (67 kDa) antibodies. (B) Endoplasmic Reticulum fractions from resting and stressed SHSY5Y were treated either with 0.05% trypsin supplemented with 1 millimol/L EDTA (Trypsin) or with 1 millimol/L EDTA alone (Ctrl). Reaction mixtures were then analyzed by Western blotting. Filters were probed with a anti-IDE (110 kDa) polyclonal antibody, with a anti-calnexin antibody (90 kDa) and with a anti-Erp57 antibody (57 kDa).

To further strengthen this evidence, sub-cellular fractionation assays were performed in resting and heat-stressed SHSY5Y. Total lysates, ER fraction and cytosolic fraction were then purified and assayed through Western blotting to get semi-quantitative insights on IDE content. Fig 6A clearly indicates that in the total lysate IDE expression increased with respect to resting cells after 24 h of recovery from heat stress, consistently with results reported in our previous paper [22]. Analysis on the purified cytosolic fraction (Fig 6A) further reinforced the evidence that heat-exposition brought about a decrease in IDE, similarly to what observed in crude cell extracts (Fig 4B). Then, the analysis in the ER fraction (Fig 6A), highlighted a consistent accumulation of IDE at 24 h of recovery from heat-stress, thus confirming the confocal microscopy outcome (Fig 5A). Moreover, filters were probed with an anti-calnexin antibody to specifically stain the ER fraction and, as expected, the analysis revealed that the ER marker was abundantly represented exclusively in the ER fraction, with minimal contamination of the cytosolic fraction (Fig 6A).

Thereafter, to get some preliminary insights on the localization of IDE in the endoplasmic reticulum *i*.*e*. endoluminal or at the cytosolic interface, a limited proteolysis by trypsin of the ER fraction was performed. To this purpose, an aliquot of ER fractions from resting and heat-stressed cells at 24 h of recovery, when a peak in IDE accumulation in such a compartment was observed (Fig 6A), were incubated either with 0.05% trypsin supplemented with 1 mmol/L EDTA or with 1 mmol/L EDTA alone, as an internal control. Then, samples were harvested at the end of the reaction and analyzed by Western blotting to address the IDE accessibility to proteolytic cleavage.

Fig 6B clearly indicates that upon trypsin treatment IDE undergoes proteolytic cleavage with the appearance of several lower molecular weight fragments (100–65 kDa). Furthermore, the effect appeared to be more pronounced in fraction from stressed cells, even though this behaviour might simply reflect a relatively higher abundance of IDE under such experimental conditions.

Antibodies directed against the luminal domain of calnexin, an ER transmembrane protein, and Erp57, an endoluminal protein, were used as positive and negative control respectively. As expected, digestion of ER fractions revealed a ≈70 kDa proteinase-resistant fragment corresponding to calnexin deprived of its cytosolic domain whereas Erp57 was fully resistant to trypsin digestion (Fig 6B).

### Decreased IDE-proteasome interaction during the HSR

The bulk of data previously discussed suggest that IDE might represent a macromolecular inhibitor of 26S proteasome activity. Further, we report that, during the HSR, the activity of the cytosolic pool of 26S proteasome is progressively rescued by the cell: interestingly, a maximum recovery is achieved at a time-point (*i*.*e*., 24 h) when IDE content is significantly decreased in the cytosol (Fig 4A), being gathered in the outer face of the ER (Figs 5 and 6).

Hence, we wondered whether the greater activity of the 26S proteasome from heat stressed cells at this time point might follow a decreased extent of binding by IDE and, thus, a reduced inhibition.

To address this task, proteasome particles from the cytosolic fraction of resting and heat stressed SHSY5Y (at 24 h of recovery) were affinity purified and analyzed by Western blotting to get a semi-quantitative comparison of the amount of IDE bound to the proteasome.

**Fig 7:**
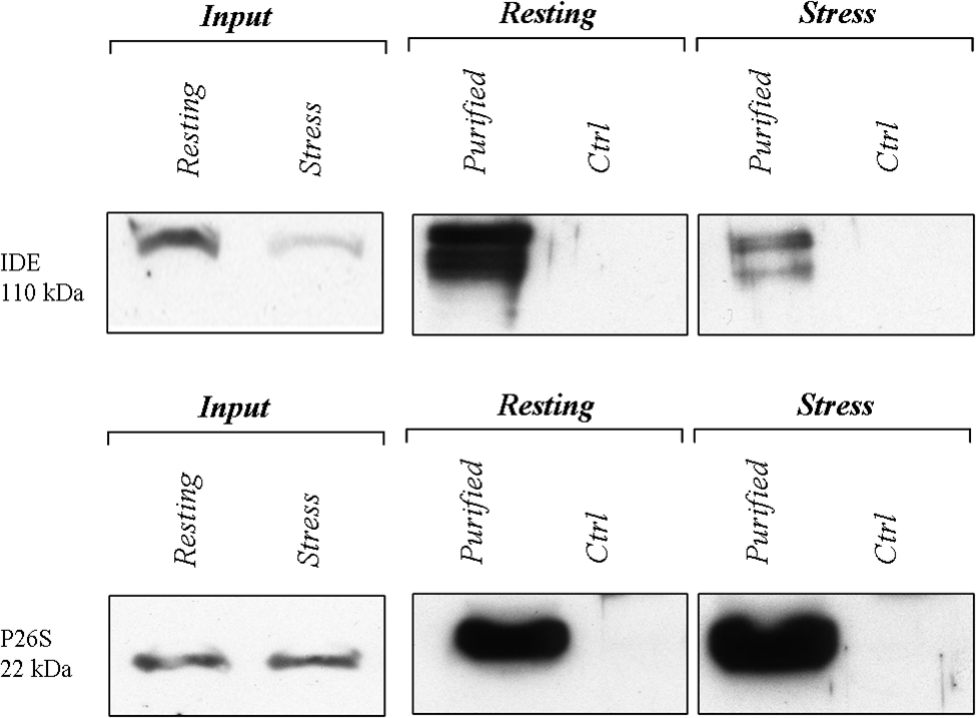
Decreased IDE-proteasome interaction during the HSR. A semi-quantitative comparison of IDE association with the proteasome by Western blotting. Membranes were stained with an anti-IDE antibody (upper panel) and with an anti-protesaome (p27 subunit) antibody (lower panel). Input, stands for 5 micrograms of cytosolic extract loaded in the gel, whereas Ctrl stands for incubation of cell lysates with non-specific beads. A representative immunoblot of three independent experiment is shown. Proteasome particles from the cytosolic fraction of resting and heat-stressed SHSY5Y were affinity purified. Notably, a lower molecular weight species is recognized by the anti-IDE antiboby. Anyway, we are unsure about the identity of this band which, anyway, is constantly observed by us and by other authors when large amount of cell extracts is loaded.

Fig 7 indicates that proteasome particles purified from heat stressed cells display a several fold lower content in IDE with respect to that purified from resting cells. As expected, no purification of proteasome is obtained by using not-specific beads, and equal quantities of purified proteasome were loaded into the gel. Staining of inputs lanes clearly confirm that no variation in the proteasome content is observed for the two experimental conditions, whereas a consistent decrease of IDE in the cytosolic fraction is confirmed to occur.

## Discussion

Herewith, by adopting different approaches, we report, for the first time, that the 26S proteasome activity is significantly affected by variations in the intracellular distribution of IDE, thus allowing to hypothesize that the IDE-proteasome interaction might represent a novel pathway of regulation of intracellular proteolysis. Furthermore, these results strengthen the largely envisaged notion that IDE biological role is of general significance, in accord with its evolutionary conservation and ubiquitous distribution. Notably, the results here reported are consistent with several intriguing observations which have emerged over the last decade, namely: *i)* IDE selects the substrates on the basis of their secondary/tertiary structure and targets amyloidogenic proteins envisaging a role as “dead-end chaperone” [57]; *ii)* IDE binds to the ubiquitin monomer [19,20]; *iii)* IDE processes sets of antigenic peptides for class I HLA presentation [17]; *iv*) IDE regulates yeast proliferation under proteotoxic conditions [15]. The overall bulk of data and the recently discussed HSP-like behaviour of IDE [22] would indeed account for its widespread contribution to the Ubiquitin-Proteasome-System (USP) in regulating key aspects of cell life.

Herewith we demonstrated that IDE down-regulation in SHSY5Y cells brings about an increase in the proteolytic activity of the 26S proteasome (Fig 2) which is effective on fluorogenic substrates as well as on poly-ubiquitinated proteins.

Moreover, the IDE inhibitory effect on 26S proteasome was also confirmed by performing an *in vitro* inhibition assay with the two purified macromolecular players (*i*.*e*. IDE and 26S proteasome) (Fig 3). Furthermore, even though we cannot at this stage obtain a deeper information on the equilibrium properties regulating this interaction, the observation that IDE silencing brings about an effect closely similar to that corresponding to a lowering of IDE concentration in enzymatic assays (Figs 2 and 3) indeed suggests that the IDE-26S proteasome complex is reversible with a very high affinity.

It is interesting to outline that the absence of insulin in our cellular cell silencing system rules out that **t**he observed effects could be referable to insulin or insulin fragments produced by IDE catalysis, which were reported to modulate proteasome activity [48,49]. Therefore, we believe that a major novelty element, which emerges from the analyses herewith discussed, is that IDE could represent a critical 26S proteasome regulator even in absence of its substrates. Intriguingly, preliminary results suggest the possibility that the IDE-dependent modulation of the 26S proteasome activity might occur through extra-catalytic mechanisms, rendering of great interest further investigations on the mechanistic insights of the interaction.

Moreover, the novel observation that IDE efficiently degrades the Z-LLE-AMC fluorogenic substrate, considered to be specific for the caspase-like activity of the proteasome, raises the question of a careful consideration when assessing the 26S proteasome caspase-like proteolysis rate.

On the basis of the recently discussed HSP-like behaviour of IDE [22], we can then formulate the reasonable hypothesis that the IDE-26S proteasome functional interaction should be critical also during the HSR.

In fact, our results demonstrate that during the HSR, the ordered and chronologically defined pattern of activity of cytosolic 26S proteasome particles observed in *wild-type* SHSY5Y (which is consistent with data from other authors, see refs. [38,47]) was lost in IDE-depleted and stressed cells, but rescued by the addition of tailored concentrations of exogenous IDE. Interestingly, the overlapping behaviour of poly-ubiquitinated proteins between *wild-type* and IDE-silenced cells (Fig 4) (with the exception of samples from unstressed cells, wherein a minor content in the last conditions was confirmed to occur) along with the evidence that the 26S proteasome content does not vary either during the HSR or upon IDE-silencing (Fig 4) (accordingly with the notion that deletion of the heat shock transcription factor (*i*.*e*. Hsf1) gene does not impair proteasome expression in stressed cells [58]) allow to hypothesize that IDE role could be restricted to the modulation of the 26S proteasome activity without affecting pathways that are upstream to the proteolytic removal of its substrates. The intrinsic complexity of the biological process, *i*.*e*. the HSR, the heterogeneity in proteasome subpopulations and the bio-availability of modulators indeed represent critical topics in determining the observations herewith reported and IDE could be one of the player.

However, the evidence that the cytosolic 26S proteasome activity at later phases of the HSR is rescued concurrently with a decreased interaction with IDE (Fig 7), likely determined by the enzyme gathering at the outer surface of the ER (Figs 5 and 6), raises the intriguing perspective, which can be only considered a working hypothesis at this stage, that the inhibitory role of IDE might be a tightly controlled pathway for the regulation of the overall proteasome activity and intracellular homeostasis.

In addition, it seems to come out that IDE migration to the ER during the HSR is a relevant aspect of the physiological role played by this ubiquitous enzyme in the regulation of cellular homeostasis. Notably, previously reported RT-PCR results clearly indicated that IDE transcription is quickly triggered upon stress exposure, turning back to basal levels within 5 h of HSR [22]: this observation appears to rule out the hypothesis that ER accumulation of IDE relies exclusively in *de novo* synthesis of the enzyme, leading instead to strengthen the idea that post-traslational modification or interaction with a still unknown molecular player might actually take place.

In conclusion, our work provides novel insight into the modulatory role of IDE on the enzymatic activities of the 26S proteasome. Notably, the regulation takes place even at nanomolar IDE concentrations and it appears reversible, as indicated by the reverse effect induced by IDE silencing, suggesting that it could represent a novel critical pathway of intracellular proteolysis regulation.

## Supporting Information

S1 Fig IDE effect on the Suc-LLVY-AMC, Boc-LLR-AMC and Z-LLE-AMC fluorogenic substrates. (A) 50 micromol/L Suc-LLVY-AMC and (B) 50 micromol/L Boc-LLR-AMC, specific for chymotryptic-like and the tryptic-like activity of the 26S proteasome, respectively, were incubated in the presence of 30 nM IDE in the 26S assay buffer (20 millimol/L Tris-HCl, 10 millimol/L MgCl_2_, 10% glycerol, 2 millimol/L DTT, 1 millimol/L ATP, pH 7.8). IDE does not cleave these fluorogenic substrates. A representative experiment is shown (C). Double reciprocal plots of Z-LLE-AMC degradation by IDE. The fluorogenic substrate was incubated in the presence of 30 nanomol/L IDE in the 26S assay buffer and catalytic parameters were calculated and reported in this work. Results presented are the means +/- S.E. of three independent experiments.
(DOC)

S2 Fig IDE effect on 26S activity in the presence of EDTA. 7.5 picomoles of IDE were incubated 45 min at 37°C in the presence of 5 millimol/L EDTA to get full inactivation of the enzyme. Thereafter, the reaction volume was diluted in the assay buffer (20 millimol/L Tris-HCl, 10 millimol/L MgCl_2_, 10% glycerol, 2 millimol/L DTT, 1 millimol/LM ATP, pH 7.8) contatining 1 nanomol/L 26S proteasome. After dilution IDE concentration was 30 nanomol/L. The reaction mixture was, then, incubated 20 min at 37°C. Finally 50 micromol/L Suc-LLVY-AMC was added and the rate of hydrolysis of the fluorogenic substrate was monitored. As internal control the activity of the 26S proteasome alone was recorded in presence of EDTA.The final concentration of EDTA in the reaction mixtures for the 26S proteasome in presence and absence of IDE were identical. As shown, the modulator effect of IDE on proteasome activity is detected also in the presence of EDTA. 26 S proteasome (—–); 26S proteasome in presence of EDTA (—–); 26S proteasome + 30 nanomol/L IDE (—–); 26S proteasome + 30 nanomol/L IDE in presence of EDTA (—–).
(DOC)

S3 Fig Analysis of IDE-26S proteasome association through Confocal Microscopy and Immuno-Precipitation. (A) Control and heat-stressed fixed SHSY5Y cells were co-stained with anti-IDE and anti-26S proteasome antibodies followed by fluorescein anti-rabbit (green) and rhodamine-red X anti-mouse (red) secondary antibodies. Merged images show a diffuse cytosolic co-localization of IDE and 26S proteasome both in control and in stressed cells. Western blotting analysis of co-immunoprecipitates from heat-stressed (B) and resting (C) SHSY5Y reveal the association of IDE with the 26S proteasome. No detectable level of IDE and 26S proteasome were obtained upon co-immunoprecipitation with non-specific IgG. It is noteworthy that result shown in panel C is in apparent contradiction with that of Fig 7. Anyway, it has to be considered that the Co-IP experiment was performed on a whole lysate. Then we hypothesize that upon dissolution of the ER, IDE is readily available for again binding to the proteasome, an event which is likely to occur also considering the apparent very high affinity for the complex formation.
(DOC)

S4 Fig Semi-quantitative analysis of IDE in the pellet fraction. The membrane-enriched pellet separated during crude cell extracts preparation (those discussed in Fig 5B) from wild type and IDE-silenced SHSY5Y cells were dissolved in standard lysis buffer supplemented with 1% Triton. The protein concentration was normalized through Bradford assay. A Western blotting analysis was then performed and filters were probed with a polyclonal anti-IDE antibody. As internal control filters were probed with a anti-Na^+^/K^+^ ATP-ase.
(DOC)
