# Supplementary figures and images for: Proteasome Activity Is Affected by Fluctuations in Insulin-Degrading Enzyme Distribution
Source: PLoS One. 2015 Jul 17;10(7):e0132455. doi: 10.1371/journal.pone.0132455 (PMC4506093; doi:10.1371/journal.pone.0132455)

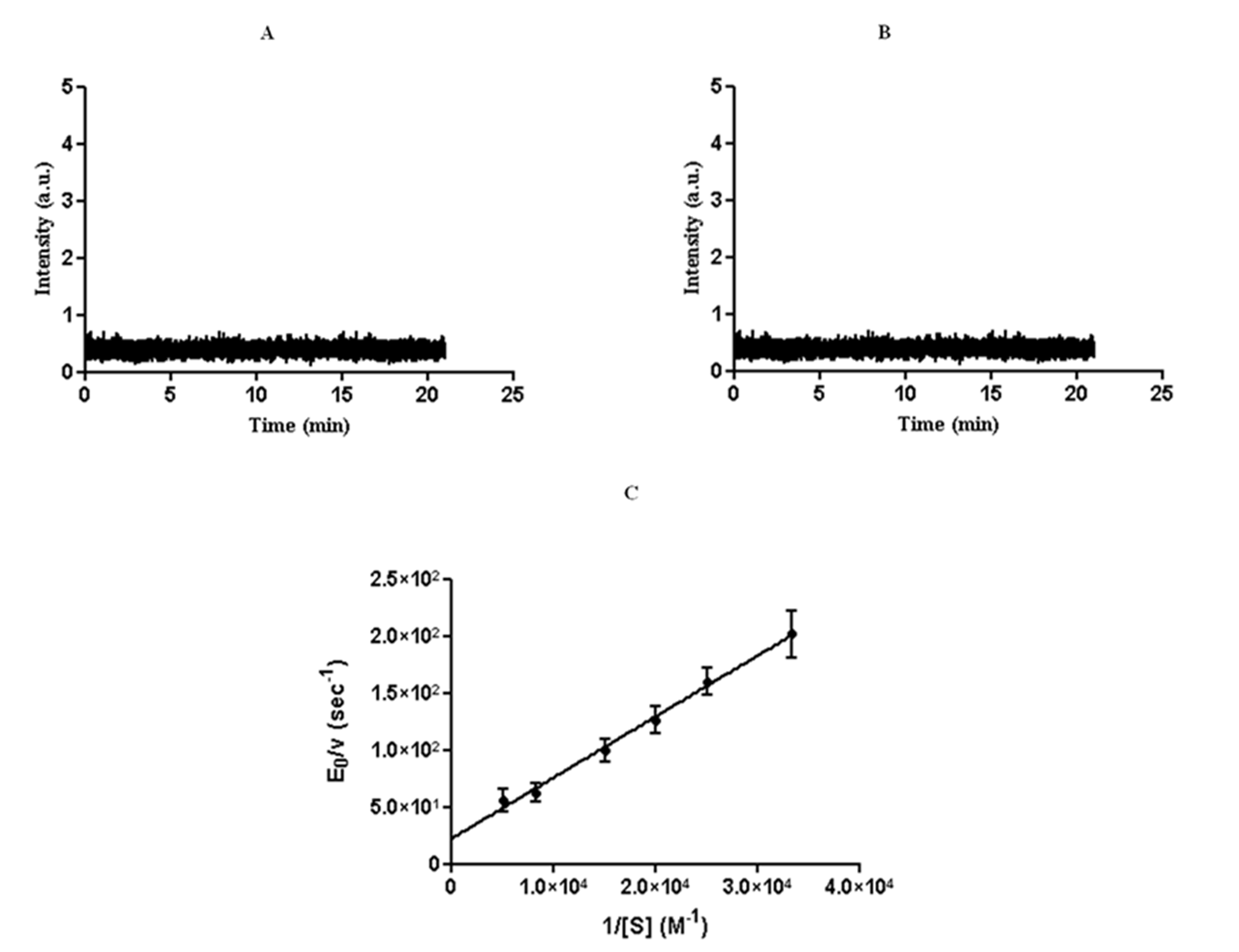


S1 Fig.

Supplement: S1 Fig — (A) 50 micromol/L Suc-LLVY-AMC and (B) 50 micromol/L Boc-LLR-AMC, specific for chymotryptic-like and the tryptic-like activity of the 26S proteasome, respectively, were incubated in the presence of 30 nM IDE in the 26S assay buffer (20 millimol/L Tris-HCl, 10 millimol/L MgCl2, 10% glycerol, 2 millimol/L DTT, 1 millimol/L ATP, pH 7.8). IDE does not cleave these fluorogenic substrates. A representative experiment is shown (C). Double reciprocal plots of Z-LLE-AMC degradation by IDE. The fluorogenic substrate was incubated in the presence of 30 nanomol/L IDE in the 26S assay buffer and catalytic parameters were calculated and reported in this work. Results presented are the means +/- S.E. of three independent experiments. (DOC) [file pone.0132455.s001.doc]

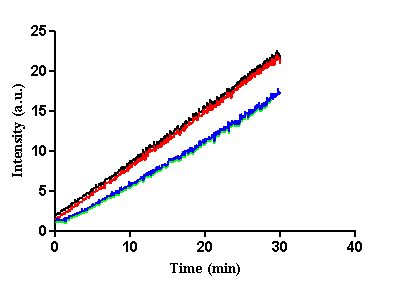


S2 Fig.

Supplement: S2 Fig — 7.5 picomoles of IDE were incubated 45 min at 37°C in the presence of 5 millimol/L EDTA to get full inactivation of the enzyme. Thereafter, the reaction volume was diluted in the assay buffer (20 millimol/L Tris-HCl, 10 millimol/L MgCl2, 10% glycerol, 2 millimol/L DTT, 1 millimol/LM ATP, pH 7.8) contatining 1 nanomol/L 26S proteasome. After dilution IDE concentration was 30 nanomol/L. The reaction mixture was, then, incubated 20 min at 37°C. Finally 50 micromol/L Suc-LLVY-AMC was added and the rate of hydrolysis of the fluorogenic substrate was monitored. As internal control the activity of the 26S proteasome alone was recorded in presence of EDTA.The final concentration of EDTA in the reaction mixtures for the 26S proteasome in presence and absence of IDE were identical. As shown, the modulator effect of IDE on proteasome activity is detected also in the presence of EDTA. 26 S proteasome (—–); 26S proteasome in presence of EDTA (—–); 26S proteasome + 30 nanomol/L IDE (—–); 26S proteasome + 30 nanomol/L IDE in presence of EDTA (—–). (DOC) [file pone.0132455.s002.doc]

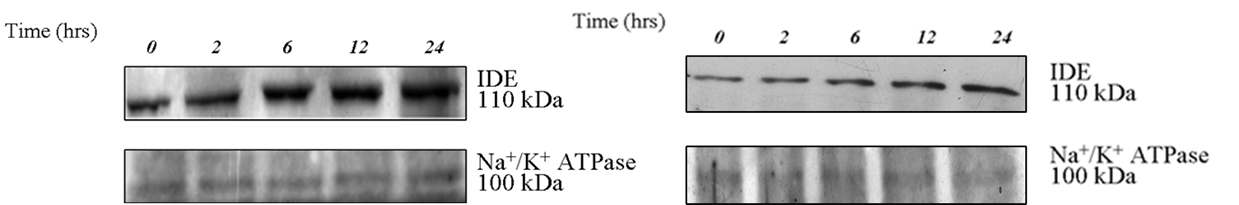


S4 Fig.

Supplement: S4 Fig — The membrane-enriched pellet separated during crude cell extracts preparation (those discussed in Fig 5B) from wild type and IDE-silenced SHSY5Y cells were dissolved in standard lysis buffer supplemented with 1% Triton. The protein concentration was normalized through Bradford assay. A Western blotting analysis was then performed and filters were probed with a polyclonal anti-IDE antibody. As internal control filters were probed with a anti-Na+/K+ ATP-ase. (DOC) [file pone.0132455.s004.doc]
